# Gender Differences in Body Evaluation: Do Men Show More Self-Serving Double Standards Than Women?

**DOI:** 10.3389/fpsyg.2019.00544

**Published:** 2019-03-12

**Authors:** Mona M. Voges, Claire-Marie Giabbiconi, Benjamin Schöne, Manuel Waldorf, Andrea S. Hartmann, Silja Vocks

**Affiliations:** ^1^Department of Clinical Psychology and Psychotherapy, Institute of Psychology, Osnabrück University, Osnabrück, Germany; ^2^Department of Experimental Psychology I, Institute of Psychology, Osnabrück University, Osnabrück, Germany

**Keywords:** body evaluation, men, women, gender difference, double standards

## Abstract

Generally speaking, compared to women, men are less dissatisfied with their own body and consider themselves to be better-looking and less overweight. So far, however, it is unclear whether these divergent body ratings arise from the application of double standards. With the present study, we examined whether men apply different standards to their own body than to other men’s bodies and whether they differ from women in this regard. To this aim, we presented *n* = 104 women and *n* = 93 men with pictures of thin, average-weight, overweight, athletic and hypermuscular male and female bodies on a computer screen. To manipulate identification, we showed the bodies of the respective participant’s gender once with the participant’s own face and once with the face of another person. Identity cues, such as faces, might activate different body schemata, which influence body ratings and thus lead to the application of double standards. Participants were instructed to rate their emotional reaction to the bodies according to valence and arousal, and to rate the bodies with respect to attractiveness, body fat, and muscle mass. The application of double standards was determined by calculating the difference between the rating of a body presented with the participant’s face and the rating of the same body presented with another person’s face. Both women and men showed self-deprecating double standards in valence, body attractiveness, body fat and muscle mass for the overweight body. Men also revealed self-deprecating double standards for the thin, average-weight and hypermuscular bodies, but evaluated the athletic body as more attractive and with a higher positive feeling when it was presented with their own face. Women did not show any self-serving double standards and showed fewer self-deprecating double standards than men. The results indicate that men devalue non-ideal bodies and upvalue ideal bodies when they are self-related, whereas women more rate in a fair-minded manner. Thus, in contrast to women, an advantage for men may be that they are able to self-enhance in the case of desirable bodies. This ability to self-enhance regarding desirable features might be beneficial for men’s self-worth and body satisfaction.

## Introduction

Body image research has mainly focused on women (McCabe and Ricciardelli, 2004), as they are more dissatisfied with their own body and more likely to develop eating disorders compared to men (Keski-Rahkonen and Mustelin, 2016; Karazsia et al., 2017). The media and society convey a thin ideal for women’s bodies, which is internalized by women and men in Western societies (Dittmar et al., 2000; Crossley et al., 2012). Women often experience a discrepancy between their own body and the - often difficult to achieve - ideal female body, leading to the emergence of body dissatisfaction (Grossbard et al., 2011). In the last few decades, however, the ideal male body has also attracted attention. In Western societies, the ideal of a more lean, muscular and V-shaped male body has developed (Ridgeway and Tylka, 2005; Smolak and Murnen, 2008; Crossley et al., 2012), as reflected in photographs in magazines (Law and Labre, 2002) or action toys (Pope et al., 1999). Nowadays, therefore, men are also confronted with an ideal body that is difficult to achieve. Accordingly, various studies have found that men, like women, feel greater dissatisfaction when they are confronted with ideal body stimuli of their own sex (Arbour and Martin Ginis, 2006; Blond, 2008; Grabe et al., 2008; Karazsia and Crowther, 2009; Galioto and Crowther, 2013; Cordes et al., 2017). Moreover, a study examining eye movements on body stimuli found that women and men show longer and more frequent attention toward bodies representing the ideal compared to other bodies. This viewing pattern might provoke body dissatisfaction in everyday life (Cho and Lee, 2013).

In contrast to these similar results for women and men, studies also suggest differences between the two sexes in terms of body image. Even in childhood, girls are already more conscious about how their body weight affects their appearance compared to boys (Shriver et al., 2013). Furthermore, girls’ body esteem is already reduced when they are overweight, whereas boys’ body esteem is only affected when they are obese (Shriver et al., 2013). A longitudinal study showed that in adolescence, body dissatisfaction increases with time in both sexes, but the highest levels of boys’ body dissatisfaction were only as high as the lowest levels of girls’ body dissatisfaction (Bucchianeri et al., 2013). In line with this, girls were found to place more emphasis on aesthetic values and less emphasis on functional values of their bodies compared to boys, and reported more dissatisfaction with both values than did boys (Abbott and Barber, 2010). This pattern of more pronounced body dissatisfaction in women than in men, and the greater influence of body weight on body image in women than in men, persists in adulthood (Algars et al., 2009). Men assess themselves as better-looking (Feingold and Mazzella, 1998) while women consider themselves as more overweight and want to lose more body weight (Lemon et al., 2009). Indeed, in a study in which most men were effectively overweight and most women were effectively average-weight or thin, the men still considered themselves as lighter than they were and the women still saw themselves as heavier than they were (McCreary and Sadava, 2001).

Various factors have been discussed as potentially contributing to the differences in body image between women and men. It has been suggested that the pressure to conform to the body ideal and to look good is higher in women than in men because women are more frequently confronted with ideal bodies in the media and because in Western societies, beauty is more essential to the feminine than to the masculine gender role (Fredrickson and Roberts, 1997; Knauss et al., 2007; Buote et al., 2011). Accordingly, body size seems to be a more relevant factor for the self-worth of women than for the self-worth of men (Owens et al., 2010). Moreover, the standards for the female body ideal depicted in society seem to be clearer, while the male body ideal comprises more divergent body types, represented by more heterogeneous, also average-weight, media images of men (Buote et al., 2011). Buote et al. (2011) also found that although women know that the ideal thin body is hard to achieve for women in general, they believe that such a body should be attainable for them personally. Men, by contrast, did not show different standards for themselves than for other men. Thus, the lower levels of body dissatisfaction in men than in women might also be evoked by the application of double standards, which are observable when different sets of requirements are applied to different persons when evaluating them (Foschi, 1996). In line with body image theory (Williamson et al., 2004), the application of double standards might suggest that self-related body stimuli activate different body schemata than other-related body stimuli, leading to different ratings of these body stimuli (Voges et al., 2017).

The application of double standards has already been observed with regard to ratings of performance: Usually, men estimate themselves as more intelligent than do women (Furnham, 2001). In a study of biology students, men and women applied the same standards to evaluate the intelligence of another student, but men considered themselves to be more intelligent than 66% of their classmates, while women only considered themselves to be more intelligent than 54% of their classmates, after controlling for prior academic ability (Cooper et al., 2018). This finding suggests that women are more likely to consider themselves as being of average intelligence, whereas men are more likely to believe that they are outstanding. These self-serving ratings in men might be evoked by double standards between one’s own intelligence and that of others (Foschi, 1996), which women do not show. Stereotypes that men have to be confident, independent and self-focused and women should be more other-oriented and modest (Moss-Racusin et al., 2010) might facilitate men’s overestimation of their own competences and characteristics in contrast to women.

In a similar vein, findings that men evaluate their own body in a more self-serving way than do women (McCreary and Sadava, 2001; Lemon et al., 2009) might also be attributable to double standards, leading men to rate their own body more positively than other bodies and women to rate their own body more critically than other bodies (Buote et al., 2011). In a recent study, we examined whether women apply double standards when evaluating body stimuli depending on the identity of the body (Voges et al., 2017). We found that women rated overweight bodies as less attractive, with more body fat and with less muscle mass, when they were presented with their own face compared to when they were presented with another woman’s face. Thus, women applied a stricter standard to themselves in the case of overweight bodies. For thin, average-weight, athletic and hypermuscular bodies, women applied the same standards for both identities, indicating no double standards in general (Voges et al., 2017). In contrast, women with anorexia nervosa and bulimia nervosa showed more pronounced self-deprecating double standards across all body types (Voges et al., 2018). Thus, women without an eating disorder diagnosis seem to mostly be fair-minded when rating their own body and other bodies, but when eating pathology exists, women rate in a self-deprecating manner (Voges et al., 2018).

With the present study, we wanted to examine whether men differ from women in the application of double standards in body evaluation depending on the body’s identity. If this is the case, double standards might be one factor contributing to the more pronounced body satisfaction in men than in women (Stanford and Lemberg, 2012). For this purpose, we presented men and women with pictures of bodies, once with their own face and once with another person’s face, in order to evaluate whether identity influences body ratings and whether there are differences between men and women in this regard. Participants reported their emotional response to the stimuli in terms of valence and arousal and rated the bodies according to body attractiveness, body fat and muscle mass. We hypothesized that men would show more self-serving double standards than women, reflected by more positive ratings of a body with one’s own face compared to a body with another person’s face. Furthermore, from an exploratory perspective, we examined how body dissatisfaction is associated with the extent of double standards in men and women (Voges et al., 2018).

## Materials and Methods

### Participants

The inclusion criteria for the female and male participants were age between 18 and 30 years and the absence of a mental disorder based on self-report with two yes/no questions (“Do you currently suffer from a diagnosed mental disorder?” “Are you currently in treatment because of a diagnosed mental disorder?”). Furthermore, only men with a Body Mass Index (BMI) of 21–26 kg/m^2^ and only women with a BMI of 18.5–23.5 kg/m^2^ were included. The World Health Organization defines average weight as a BMI of 18.5–24.9 kg/m^2^ for both genders (World Health Organization, 2019). However, as BMI is influenced by age and gender (Nevill and Metsios, 2015) and is typically higher in men than in women in Western societies (Kromeyer-Hauschild et al., 2001; Finucane et al., 2011; Statistisches Bundesamt (Destatis), 2017), we defined a higher BMI range for men, but ensured that the same range of 5 BMI points was employed for both genders. For a non-athletic college population, cutoffs for overweight were defined as 24.0 kg/m^2^ for women and 26.5 kg/m^2^ for men (Ode et al., 2007). We set our BMI limits in order to examine average-weight women and men who are most representative for their age and have the greatest resemblance to the average-weight body used in our study as stimulus material.

Participants were recruited via university lectures, press releases, Facebook advertisements or flyers. We measured *N* = 109 men, 14 of whom were excluded because they did not fulfill the BMI or age criterion. The female participants were *N* = 104 women from our previous study (Voges et al., 2017). Group characteristics and statistics for group comparisons are depicted in Table 1. The women were slightly younger than the men and had a lower BMI. Furthermore, women reported more eating concerns, weight concerns, shape concerns, body dissatisfaction and drive for thinness than men, as measured by the Eating Disorder Examination Questionnaire (EDE-Q) and the Eating Disorder Inventory (EDI-2).

**Table 1:** Group comparisons of women and men regarding age, body mass index, eating pathology, and body image disturbance.

	Women (*n* = 104)		Men (*n* = 93)		Test statistics		
Variables	*M*	*SD*	*M*	*SD*	*t*	*df*	*p*
Age (years)	21.29	2.84	23.13	2.98	-4.44	195	<0.001
Body mass index (kg/m^2^)	20.79	1.25	23.12	1.26	-13.03	195	<0.001
**Eating Disorder Examination Questionnaire (EDE-Q)**					
Restraint	0.89	1.02	0.86	1.12	0.19	195	0.853
Eating concern	0.53	0.68	0.26	0.47	3.24	184.52	0.001
Weight concern	1.17	1.10	0.63	0.58	4.39	159.29	<0.001
Shape concern	1.53	1.10	1.05	0.91	3.36	193.68	0.001
**Eating Disorder Inventory-2 (EDI-2)**					
Body dissatisfaction	2.90	0.97	2.29	0.78	4.88	192.98	<0.001
Drive for thinness	2.38	1.02	1.68	0.65	5.78	176.26	<0.001


**T*-tests or (if indicated by Levene’s tests) Welch-tests were conducted. *M*, Mean; *SD*, standard deviation; *df*, degrees of freedom.*

### Questionnaires

Eating pathology was measured by the German version of the EDE-Q (Fairburn and Cooper, 1993; Hilbert and Tuschen-Caffier, 2016), which comprises the four subscales Restraint, Eating concern, Weight concern and Shape concern. The EDE-Q contains 22 items, which are rated on a 7-point Likert scale from *no days/not at all* (0) to *every day/markedly* (6) and refer to the last 28 days. Internal consistencies (Cronbach’s α) for women and men, respectively, in the current sample were α = 0.83 and α = 0.77 for Restraint, α = 0.79 and α = 0.60 for Eating Concern, α = 0.81 and α = 0.46 for Weight Concern, and α = 0.89 and α = 0.82 for Shape Concern. Overall, the internal consistencies were acceptable, except for Eating Concern and Weight Concern in men. Previous studies on male norms for the EDE-Q also showed lower reliability scores in men than in women (Reas et al., 2012). The authors suggested that this might be a result of inconsistent responding in men, less relevant or less appropriate items for men, or a floor effect in men. In line with the latter possible reason, the men in our sample showed very low means and standard deviations on Eating Concern and Weight Concern.

Body dissatisfaction and drive for thinness were measured by the two subscales of the same name from the German version of the EDI-2 (Garner, 1991; Paul and Thiel, 2005). Drive for thinness comprises seven items and Body dissatisfaction comprises nine items, which are rated on a 6-point Likert scale ranging from *never* (1) to *always* (6). In the current sample, Cronbach’s α for women and men, respectively, were α = 0.90 and α = 0.77 for Drive for thinness and α = 0.88 and α = 0.81 for Body dissatisfaction.

### Stimulus Material

**FIGURE 1:**
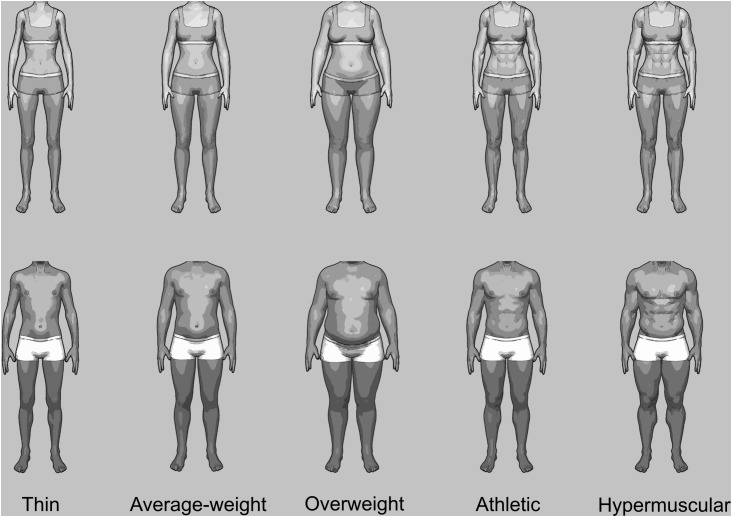
The female and male thin, average-weight, overweight, athletic and hypermuscular bodies.

To create the stimulus material, for each sex, we generated five types of body build, i.e., thin, average-weight, overweight, athletic and hypermuscular, using the software DAZ studio 4.6 (DAZ Productions, Inc.; Salt Lake City, UT, United States). Every body build comprised bodies in five poses, resulting in 25 female bodies and 25 male bodies. To manipulate identification with these bodies, we took a photo of each participant’s head with a neutral facial expression and in frontal view, using a Panasonic Lumix DMC-TZ8 digital camera. To place the heads of the participants on the bodies, the faces were cut out (along with the hair), mirror-imaged, and then placed on the bodies using MATLAB 2008 (MathWorks; Natick, MA, United States). To create homogenous body stimuli from the faces and bodies, we gray-scaled the final stimuli and applied a comic filter using AKVIS ArtWork 8.1 (AKVIS; Perm, Russia). Men’s faces were only placed on male bodies and women’s faces were only placed on female bodies. The same manipulation procedure was also conducted with another female face and the 25 female bodies as well as with another male face and the 25 male bodies. The faces for the other-condition were taken from the CAL/PAL Face Database (Minear and Park, 2004) and chosen because they were rated as averagely attractive (labeled “neutral_y_f_25” and “neutral_y_m_22”) (Ebner, 2008). In total, we presented every man with 75 body stimuli encompassing 25 female bodies with a woman’s face (i.e., the face used in the other-condition for the women), 25 male body stimuli with one’s own face, and the identical 25 male body stimuli with the other man’s face. Correspondingly, every woman saw 75 body stimuli encompassing 25 male bodies with a man’s face, 25 female body stimuli with one’s own face, and the identical 25 female body stimuli with the other woman’s face. As the opposite-sex pictures made the pool of stimuli larger, it was less likely that similar bodies would follow each other. All pictures had a 1400 × 1050 px format. Figure 1 presents the pictures of the bodies without faces.

### Procedure

This study was performed in accordance with the Declaration of Helsinki and the recommendations of the Osnabrück University ethics committee. The protocol was approved by the Osnabrück University ethics committee (reference number: 4/71043.5). All subjects gave written informed consent in accordance with the Declaration of Helsinki. First, the photo of the participant’s face was taken. Next, the experimenters edited the photos and created the individual stimulus material, while the participants answered the questionnaires. At the beginning of the task, participants were instructed that after each presentation of a body stimulus, they should respond to two scales asking about the emotions they experienced when the stimulus was presented, i.e., valence from *very negative* (1) to *very positive* (9) and arousal from *very calm* (1) to *very arousing* (9). Furthermore, they were asked to respond to three scales referring to the presented body stimulus, i.e., body attractiveness from *very unattractive* (1) to *very attractive* (9), body fat from *very little body fat* (1) to *very much body fat* (9) and muscle mass from *very little muscle mass* (1) to *very much muscle mass* (9). Additionally, participants were told that in some cases, their own face would be shown on the bodies and that this should help them to identify with the bodies. All body stimuli were presented for 3 s in a randomized order and all ratings were made on Likert scales from 1 to 9 using the experimental software E-Prime^®^ 2.0 (Psychology Software Tools, Inc.; Sharpsburg, PA, United States). Finally, participants were asked once to evaluate how coherent the body stimuli looked overall, thus how well bodies and heads matched from *very poor match* (1) to *very good match* (9). The rating of coherence did not differ between women (*M* = 5.76, *SD* = 1.92) and men (*M* = 5.56, *SD* = 2.02), *t*(195) = 0.71, *p* = 0.476. All participants received student participant credit or monetary compensation (7–20 Euros).

### Statistical Analysis

Statistical analyses were conducted using SPSS Statistics (IBM; Armonk, NY, United States). To examine whether women and men classified the bodies in line with our expectations, we ran a 2 × 5 repeated measures MANOVA with the factors Group (Women, Men) and Build (Thin, Average-weight, Overweight, Athletic, Hypermuscular) and the absolute ratings of valence, arousal, body attractiveness, body fat and muscle mass for the bodies presented with one’s own face as dependent variables. To analyze the application of double standards, we determined DS scores by calculating the differences between the rating of a body with the participant’s face and the other person’s face for each body build. Thus, a DS score of about zero would suggest no use of double standards in body evaluation, and the greater the DS score deviates from zero, the greater the extent of double standard application. To test whether the DS scores were significantly different from zero, we used 95% confidence intervals of the DS scores. To examine how participants’ gender and the build of the presented bodies affected DS scores, we calculated a 2 × 5 repeated measures MANOVA with the factors Group (Women, Men) and Build (Thin, Average-weight, Overweight, Athletic, Hypermuscular) and the DS scores of valence, arousal, body attractiveness, body fat and muscle mass as dependent variables. In the case of significant MANOVA effects, *post hoc* ANOVAs were conducted, for which we applied the Greenhouse–Geisser correction by default and report partial eta-squared $\eta_{p}^{2}$ as a measure of effect size. For *post hoc* *t*-tests, we always report the Bonferroni-corrected *p*-values. To examine how body dissatisfaction is associated with the application of double standards, for both genders, we calculated Spearman’s Rho correlations between body dissatisfaction measured by the EDI-2 and the DS scores s of each body build.

## Results

### Absolute Ratings of the Bodies With One’s Own Face

The MANOVA yielded a significant main effect of Build, Pillai’s trace = 0.98, *F*(20,176) = 381.40, *p* < 0.001,η$\eta_{p}^{2}$ = 0.98, a significant main effect of Group, Pillai’s trace = 0.65, *F*(5,191) = 69.89, *p* < 0.001,$\eta_{p}^{2}$ = 0.65, and a significant interaction of Build × Group, Pillai’s trace = 0.84, *F*(20,176) = 44.86, *p* < 0.001,$\eta_{p}^{2}$ = 0.84. In the following, the ANOVAs and post-hoc results for each rating variable are described. Means, standard errors, and post hoc *t*-test results of the rating variables are presented in Table 2.

**Table 2:** Means, standard errors, and *post hoc* *t*-test results for each rating variable dependent on the factors Group and Build.

	Women		Men		Over both groups	
Variables	*M*	*SE*	*M*	*SE*	*M*	*SE*
**Valence**						
Thin	3.60^bde^	0.16	4.34^aef^	0.16	3.97^def^	0.11
Average-weight	6.33^bcefg^	0.12	4.42^aef^	0.13	5.38^cefg^	0.09
Overweight	2.69^cdfg^	0.13	2.70^cdfg^	0.13	2.70^cdfg^	0.09
Athletic	3.72^bdeg^	0.12	5.92^acdeg^	0.13	4.82^cdeg^	0.09
Hypermuscular	3.29^bdef^	0.13	4.59^aef^	0.14	3.94^def^	0.10
Over all builds	3.93^b^	0.07	4.40^a^	0.08	4.16	0.05
**Arousal**						
Thin	4.40	0.17	4.09^dg^	0.18	4.25^d^	0.12
Average-weight	4.07	0.15	3.69^cefg^	0.16	3.88^cefg^	0.11
Overweight	4.32	0.19	4.16^d^	0.20	4.24^d^	0.14
Athletic	4.23	0.16	4.25^d^	0.17	4.24^d^	0.11
Hypermuscular	4.35	0.17	4.45^cd^	0.18	4.40^d^	0.12
Over all builds	4.28	0.15	4.13	0.16	4.20	0.11
**Body attractiveness**						
Thin	3.71^bde^	0.17	4.35^aef^	0.18	4.03^def^	0.12
Average-weight	6.70^bcefg^	0.12	4.32^aef^	0.12	5.51^cefg^	0.09
Overweight	2.50^bcdfg^	0.10	1.97^acdfg^	0.11	2.24^cdfg^	0.08
Athletic	3.78^bdeg^	0.13	6.28^acdeg^	0.14	5.03^cdeg^	0.10
Hypermuscular	3.30^bdef^	0.15	4.62^aef^	0.15	3.96^def^	0.11
Over all builds	4.00^b^	0.08	4.31^a^	0.08	4.15	0.06
***Body fat***						
Thin	1.69^bdef^	0.07	2.69^adefg^	0.08	2.19^defg^	0.05
Average-weight	3.77^bcefg^	0.10	5.69^acefg^	0.10	4.73^cefg^	0.07
Overweight	7.60^bcdfg^	0.08	8.17^acdfg^	0.08	7.89^cdfg^	0.06
Athletic	2.08^bcde^	0.09	3.99^acdeg^	0.10	3.04^cdeg^	0.07
Hypermuscular	1.88^bde^	0.11	3.16^acdef^	0.12	2.52^cdef^	0.08
Over all builds	3.41^b^	0.05	4.74^a^	0.06	4.07	0.04
**Muscle mass**						
Thin	2.43^bdfg^	0.11	2.96^adfg^	0.12	2.69^dfg^	0.08
Average-weight	4.59^bcefg^	0.10	4.28^acefg^	0.11	4.43^cefg^	0.08
Overweight	2.41^bdfg^	0.13	2.92^adfg^	0.14	2.67^dfg^	0.09
Athletic	7.87^bcdeg^	0.08	6.47^acdeg^	0.08	7.17^cdeg^	0.06
Hypermuscular	8.21^cdef^	0.06	8.10^cdef^	0.06	8.15^cdef^	0.04
Over all builds	5.10	0.06	4.94	0.06	5.02	0.04


**M*, Mean; *SE*, standard errors. ^*a*^Differs significantly from women, ^*b*^differs significantly from men, ^*c*^differs significantly from the thin build, ^*d*^differs significantly from the average-weight build, ^*e*^differs significantly from the overweight build, ^*f*^differs significantly from the athletic build, and ^*g*^differs significantly from the hypermuscular build.*

### Valence

The ANOVA for valence revealed a significant main effect of Build, *F*(3.13,610.61) = 131.77, *p* < 0.001,$\eta_{p}^{2}$ = 0.40, a significant main effect of Group, *F*(1,195) = 19.36, *p* < 0.001,$\eta_{p}^{2}$ = 0.09, and a significant interaction of Build × Group, *F*(3.13,610.61) = 76.58, *p* < 0.001,$\eta_{p}^{2}$ = 0.28. Women experienced the most positive feelings when the average-weight body was presented, whereas men experienced the most positive feelings when the athletic body was presented. Both groups experienced the most negative feelings in the case of the overweight body. For the thin and hypermuscular bodies, women reported less positive feelings than did men.

### Arousal

The ANOVA for arousal yielded a significant main effect of Build, *F*(3.34,650.45) = 9.40, *p* < 0.001,$\eta_{p}^{2}$ = 0.05, and a significant interaction of Build × Group, *F*(3.34,650.45) = 2.65, *p* = 0.04,$\eta_{p}^{2}$ = 0.01, but no significant main effect of Group, *F*(1,195) = 0.48, *p* = 0.488,$\eta_{p}^{2}$ < 0.01. Across both groups, participants experienced less arousal for the average-weight body than for the other bodies. Furthermore, men experienced less arousal for the thin body than for the hypermuscular body.

### Body Attractiveness

The ANOVA for body attractiveness revealed a significant main effect of Build, *F*(2.91,567.67) = 191.60, *p* < 0.001,$\eta_{p}^{2}$ = 0.50, a significant main effect of Group, *F*(1,195) = 7.97, *p* = 0.005, $\eta_{p}^{2}$ = 0.04, and a significant interaction of Build × Group, *F*(2.91,567.67) = 104.53, *p* < 0.001, $\eta_{p}^{2}$ = 0.35. Women evaluated the average-weight body as most attractive, while men evaluated the athletic body as most attractive. Both groups rated the overweight body as most unattractive, which was significantly more pronounced in men than in women. For the thin and hypermuscular bodies, women rated lower body attractiveness than did men.

### Body Fat

The ANOVA for body fat yielded a significant main effect of Build, *F*(3.38,659.07) = 1579.30, *p* < 0.001, $\eta_{p}^{2}$ = 0.89, a significant main effect of Group, *F*(1,195) = 286.98, *p* < 0.001, $\eta_{p}^{2}$ = 0.60, and a significant interaction of Build × Group, *F*(3.38,659.07) = 24.68, *p* < 0.001, $\eta_{p}^{2}$ = 0.11. Across all body builds, men estimated more body fat than did women. Men estimated the most body fat for the overweight body, followed by the average-weight body, the athletic, hypermuscular and finally the thin body. Women rated the most body fat for the overweight body, followed by the average-weight body, the athletic body, and finally the hypermuscular and thin bodies, which did not differ significantly from each other.

### Muscle Mass

The ANOVA for muscle mass yielded a significant main effect of Build, *F*(3.06,596.88) = 1524.57, *p* < 0.001,$\eta_{p}^{2}$ = 0.89, and a significant interaction of Build × Group, *F*(3.06,596.88) = 36.80, *p* < 0.001,$\eta_{p}^{2}$ = 0.16, but no significant main effect of Group, *F*(1,195) = 3.64, *p* = 0.058,$\eta_{p}^{2}$ = 0.02. Women and men estimated the most muscle mass for the hypermuscular body, followed by the athletic body, the average-weight body and finally the thin and overweight bodies. Women rated less muscle mass than men for the thin and overweight bodies, while men estimated less muscle mass than women for the average-weight and athletic bodies. Ratings did not differ significantly for the hypermuscular body.

### Double Standards

**FIGURE 2:**
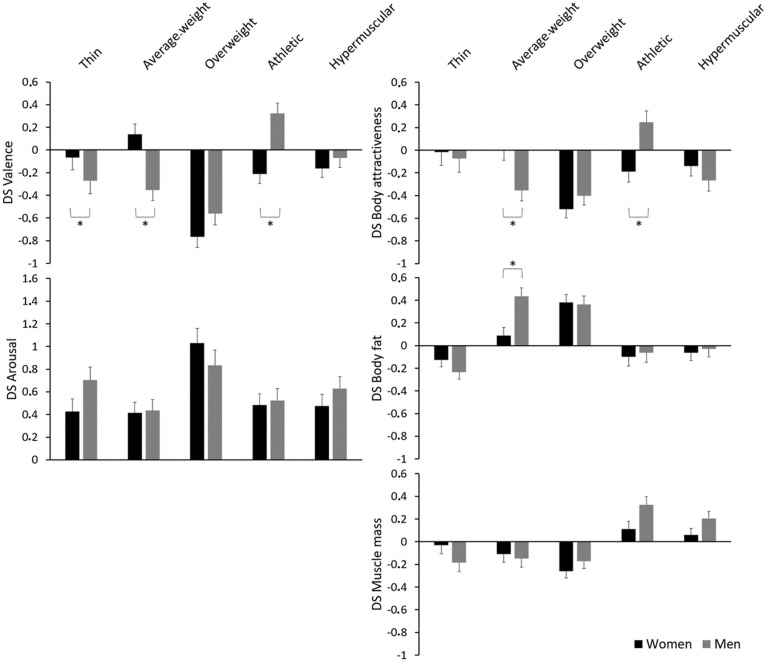
Means and standard errors (error bars) for the double standard scores (DS scores) for valence, arousal, body attractiveness, body fat and muscle mass dependent on the factors Group and Build. Asterisks highlight significant Bonferroni-corrected group differences.

The MANOVA yielded a significant main effect of Build, Pillai’s trace = 0.51, *F*(20,176) = 9.18, *p* < 0.001,$\eta_{p}^{2}$ = 0.51, and a significant interaction of Build × Group, Pillai’s trace = 0.33, *F*(20,176) = 4.25, *p* < 0.001,$\eta_{p}^{2}$ = 0.33, but no significant main effect of Group, Pillai’s trace = 0.02, *F*(5,191) = 0.66, *p* = 0.656,$\eta_{p}^{2}$ = 0.02. In the following, we report which DS scores were significant by describing the differences in body ratings depending on the faces. Furthermore, the ANOVAs and post-hoc results examining the influence of Group and Build on the DS scores are described for each DS score category. Means, standard errors, confidence intervals of the DS scores, and *post hoc* *t*-test results of the DS scores are presented in Table 3. Furthermore, Figure 2 illustrates the means, standard errors and group differences.

**Table 3:** Means, standard errors, confidence intervals of the means, and *post hoc t*-test results for the DS scores s for each rating variable dependent on the factors Group and Build.

	Women		Men		Over both groups	
Variables	*M*	*SE*	*M*	*SE*	*M*	*SE*
**DS valence**						
Thin	0.065^be^	0.111	-0.271^∗af^	0.117	-0.103^e^	0.080
Average-weight	0.140^befg^	0.089	-0.353^∗af^	0.093	-0.106^e^	0.064
Overweight	-0.765^∗cdfg^	0.094	-0.561^∗fg^	0.100	-0.663^∗cdfg^	0.069
Athletic	-0.210^∗bde^	0.087	0.323^∗acdeg^	0.091	0.056^e^	0.063
Hypermuscular	-0.163^∗de^	0.080	-0.071^ef^	0.084	-0.117^∗e^	0.058
Over all builds	-0.187^∗^	0.053	-0.187^∗^	0.056	-0.187^∗^	0.039
**DS arousal**						
Thin	0.427^∗^	0.110	0.703^∗^	0.116	0.565^∗e^	0.080
Average-weight	0.415^∗^	0.093	0.434^∗^	0.099	0.425^∗e^	0.068
Overweight	1.029^∗^	0.130	0.832^∗^	0.136	0.931^∗cdfg^	0.094
Athletic	0.485^∗^	0.099	0.523^∗^	0.104	0.504^∗e^	0.072
Hypermuscular	0.475^∗^	0.101	0.628^∗^	0.107	0.551^∗e^	0.074
Over all builds	0.566^∗^	0.077	0.624^∗^	0.081	0.595^∗^	0.056
**DS body attractiveness**						
Thin	-0.017^e^	0.116	-0.073	0.122	-0.045^e^	0.084
Average-weight	-0.002^be^	0.088	-0.355^∗af^	0.092	-0.178^∗e^	0.063
Overweight	-0.521^∗cdfg^	0.077	-0.402^∗f^	0.082	-0.462^∗cdfg^	0.056
Athletic	-0.188^∗be^	0.093	0.247^∗adeg^	0.098	0.029^e^	0.068
Hypermuscular	-0.140^e^	0.089	-0.267^∗f^	0.094	-0.204^∗e^	0.065
Over all builds	-0.174^∗^	0.055	-0.170^∗^	0.059	-0.172^∗^	0.040
**DS body fat**						
Thin	-0.127^∗e^	0.059	-0.234^∗de^	0.062	-0.181^∗de^	0.043
Average-weight	0.088^be^	0.073	0.434^∗acfg^	0.076	0.261^∗cfg^	0.053
Overweight	0.381^∗cdfg^	0.069	0.363^∗cfg^	0.073	0.372^∗cfg^	0.050
Athletic	-0.098^e^	0.081	-0.062^de^	0.086	-0.080^de^	0.059
Hypermuscular	-0.063^e^	0.068	-0.028^de^	0.072	-0.046^de^	0.049
Over all builds	0.036	0.032	0.095^∗^	0.034	0.065^∗^	0.023
**DS muscle mass**						
Thin	-0.031	0.076	-0.183^∗^	0.080	-0.107^fg^	0.055
Average-weight	-0.110	0.071	-0.148	0.075	-0.129^∗fg^	0.052
Overweight	-0.260^∗^	0.061	-0.172^∗^	0.064	-0.216^∗fg^	0.044
Athletic	0.112	0.068	0.327^∗^	0.072	0.219^∗cde^	0.049
Hypermuscular	0.060	0.057	0.206^∗^	0.060	0.133^∗cde^	0.042
Over all builds	-0.046	0.031	0.006	0.033	-0.020	0.023


*DS, double standard. *M*, Mean. *SE*, standard errors that were used for calculation of the 95% confidence interval for each DS score. ^∗^Zero is out of 95% confidence interval, ^*a*^differs significantly from women, ^*b*^differs significantly from men, ^*c*^differs significantly from the thin build, ^*d*^differs significantly from the average-weight build, ^*e*^differs significantly from the overweight build, ^*f*^differs significantly from the athletic build, and ^*g*^differs significantly from the hypermuscular build.*

### Double Standard Score in Valence

Women experienced more negative feelings when their own face was presented compared to the other person’s face in the case of the overweight, athletic and hypermuscular bodies. For men, more negative feelings emerged for one’s own face compared to the other person’s face in the case of the thin, average-weight, and overweight bodies. However, for the athletic body, men experienced more positive feelings when their own face was presented compared to the other person’s face. The ANOVA for the DS score in valence revealed a significant main effect of Build, *F*(3.59,700.03) = 20.27, *p* < 0.001,$\eta_{p}^{2}$ = 0.09, and a significant interaction of Build × Group, *F*(3.59,700.03) = 11.47, *p* < 0.001,$\eta_{p}^{2}$ = 0.06. Across both groups, the overweight body revealed more negative DS scores than all other body builds. Men showed significantly more negative DS scores than women in the case of the thin and average-weight bodies and a significantly more positive DS score than women for the athletic body.

### Double Standard Score in Arousal

For all body builds, both groups experienced more arousal when one’s own face was presented compared to the other person’s face. The ANOVA for the DS score in arousal yielded a significant main effect of Build, *F*(3.64,710.53) = 10.32, *p* < 0.001,$\eta_{p}^{2}$ = 0.05, but no significant interaction of Build × Group, *F*(3.64,710.53) = 2.09, *p* = 0.088,$\eta_{p}^{2}$ = 0.01. Across both groups, the DS score for arousal was more pronounced for the overweight body than for all other body builds.

### Double Standard Score in Body Attractiveness

Women evaluated a body as less attractive when their own face was presented compared to the other person’s face in the case of the overweight and athletic bodies. For men, a lower rating of attractiveness emerged for one’s own face compared to the other person’s face in the case of the average-weight, overweight, and hypermuscular bodies. However, men rated the athletic body as more attractive when their own face was presented compared to the other person’s face. The ANOVA for the DS score in body attractiveness revealed a significant main effect of Build, *F*(3.61,703.18) = 9.49, *p* < 0.001, $\eta_{p}^{2}$ = 0.05, and a significant interaction of Build × Group, *F*(3.61,703.18) = 5.82, *p* < 0.001,$\eta_{p}^{2}$ = 0.03. Across both groups, the overweight body resulted in more pronounced DS scores than all other body builds. Men showed a significantly more negative DS score than women for the average-weight body and a significantly more positive DS score than women for the athletic body.

### Double Standard Score in Body Fat

Women and men estimated more body fat for the overweight body and less body fat for the thin body when their own face was presented compared to the other person’s face. Furthermore, men estimated more body fat for the average-weight body when their own face was presented compared to the other person’s face. The ANOVA for the DS score in body fat yielded a significant main effect of Build, *F*(3.84,747.42) = 21.84, *p* < 0.001,$\eta_{p}^{2}$ = 0.10, and a significant interaction of Build × Group, *F*(3.84,747.42) = 2.82, *p* = 0.026,$\eta_{p}^{2}$ = 0.01. Across both groups, the average-weight and overweight bodies yielded more pronounced DS scores than the thin, athletic and hypermuscular bodies. Men showed a significantly more pronounced DS score than women in the case of the average-weight body.

### Double Standard Score in Muscle Mass

Women estimated less muscle mass for the overweight body when their own face was presented compared to the other person’s face. For men, a lower rating of muscle mass for one’s own face compared to the other person’s face was found in the case of the thin and overweight bodies. However, men estimated more muscle mass for the athletic and hypermuscular bodies when their own face was presented compared to the other person’s face. The ANOVA for the DS score in muscle mass yielded a significant main effect of Build, *F*(3.74,729.95) = 14.94, *p* < 0.001,$\eta_{p}^{2}$ = 0.07, but just failed to reach a significant interaction of Build × Group, *F*(3.74,729.95) = 2.34, *p* = 0.058, $\eta_{p}^{2}$ = 0.01. Across both groups, the DS scores for the athletic and hypermuscular bodies differed significantly from the DS scores for the thin, average-weight and overweight bodies.

### Correlations of Body Dissatisfaction and Double Standards

The higher the body dissatisfaction score of women, the less self-serving were the double standards in valence for the overweight body (*r_s_* = -0.235, *p* = 0.016) and the double standards in body attractiveness for the average-weight body (*r_s_* = -0.194, *p* = 0.048). Further correlations for women and for men were not significant, all *p* > 0.057.

## Discussion

The present study aimed to examine whether men differ from women in the application of double standards in body evaluation depending on the body’s identity. For this purpose, different body builds were presented once with the participant’s own face and once with another person’s face. The difference between the ratings of an objectively same-looking body with one’s own face and with the other face was used as a measure of the application of double standards.

In the case of an overweight body, women and men experienced more negative emotions and evaluated the body as more unattractive, with more body fat and with less muscle mass, when it was presented with their own face compared to the other person’s face. For both genders, the double standards in valence, arousal and body attractiveness for the overweight body were significantly more pronounced than for all other body builds. This clear rejection of overweight bodies might be explained by the common stereotypes in Western societies which associate obesity with being lazy or less competent, and by the stigmatization of overweight women and men (Hilbert et al., 2008; Puhl and Heuer, 2009). Accordingly, women and men evaluated the overweight body as the most unattractive. Furthermore, the identification with an overweight body might activate body schemata (Williamson et al., 2004) that being overweight oneself would be terrible, which might lead to greater fear, repulsed emotional reactions and a consequently harsher evaluation of the body (Voges et al., 2017).

In contrast to the overweight body, men showed self-serving double standards in valence, body attractiveness and muscle mass for the body rated as the most attractive, i.e., the athletic body, which mostly corresponds to the ideal body build of men (Crossley et al., 2012). Identification with the attractive athletic body might activate body schemata that having such a body would be beneficial, e.g., for mate choice (Hönekopp et al., 2007), thus leading to more positive ratings of this body. For the thin body, men rated less valence, less body fat and less muscle mass for their own face compared to the other face, which might be triggered by the body schemata that the body is too “weedy” to be attractive and desirable. Furthermore, for bodies with one’s own face, compared to the other face, men rated less valence, less body attractiveness and more body fat in the case of the average-weight body and less body attractiveness and more muscle mass in the case of the hypermuscular body. This might be linked to body schemata that the average-weight body is not sufficiently trained and that the hypermuscular body shows too much muscle mass. These bodies seem to be more acceptable for other people and less acceptable for oneself. In contrast to men, women did not show a significant self-serving double standard for the body they rated as most attractive, i.e., the average-weight body. Besides the double standards for overweight bodies outlined above, women felt fewer positive emotions in the case of the athletic and hypermuscular bodies and rated the athletic body as less attractive when their own face was presented. Similar to men, body schemata that these muscular bodies are undesirable for oneself might trigger more negative reactions for one’s own face.

To interpret these findings, it might be useful to draw on results from self-evaluation research in personality psychology. For instance, it was found that with regard to extremely desirable or undesirable traits, desirability exerts a greater influence on self-evaluations than on evaluations of others (John and Robins, 1993). In contrast, neutral traits are less influenced by the identity of the person being evaluated (John and Robins, 1993). Thus, it might be that our participants evaluated body stimuli in a more extreme manner when the stimuli were self-related (as induced by their own face) and when they were very desirable or very undesirable. In the present study, women and men experienced more arousal when their own face was presented compared to another face, which underlines the higher motivational relevance of self-related information compared to information about other persons (Bradley et al., 2001). Men in particular rated the non-ideal bodies more negatively when they were self-related and the “ideal” athletic body more positively when it was self-related. Women evaluated the “negative” overweight body as more negative when it was self-related, while they appeared to be less influenced by identity with regard to the other bodies.

Considerations from research on “fat talk” may help to explain why women and men differ in the application of double standards. Studies have found that women engage more in fat talk than do man, i.e., they more frequently talk in groups about their body dissatisfaction (Martz et al., 2009). Furthermore, they feel more pressure to engage in fat talk than men, whereas men report more pressure to engage in positive and self-accepting body talk (Martz et al., 2009). It has been suggested that it is important for women to appear friendly and agreeable and to emphasize similarities in groups in order to encourage harmony and positive feelings in a group (Britton et al., 2006). Thus, women may refrain from evaluating themselves as better than other women in order to avoid appearing selfish or conceited. This would fit with our finding that women do not evaluate bodies more positively when the bodies are presented with their own face. On the other hand, as the women in our sample were of average weight and low levels of eating pathology and body dissatisfaction, they might not evaluate bodies more critically in general when they are presented with their own face. In contrast, it was found that women with an eating disorder diagnosis showed more pronounced and more self-deprecating double standards in general than women without an eating disorder diagnosis (Voges et al., 2018). Thus, women without an eating disorder might be mostly fair-minded when evaluating female bodies, while women with eating pathology are very self-deprecating. Men, as mentioned above, feel more pressure to engage in positive and self-accepting body talk, and their body talk is more often positive than that of women (Martz et al., 2009; Engeln et al., 2013). In contrast to women, masculine stereotypes that call for men to be proud, strong, dominant and successful (Moss-Racusin et al., 2010) might facilitate men to evaluate their own body as better than the bodies of others when they believe that the body matches the ideal and thus enables them to promote themselves.

In our sample, men reported less eating pathology and body dissatisfaction than did women, which is consistent with previous studies (Stanford and Lemberg, 2012). As men did not evaluate every body build more positively or as more attractive when the body was presented with their own face, it does not appear to be true that men generally see their bodies “through rose-colored glasses.” Furthermore, it does not seem to be the case that men do not care about how their own body looks; rather, they appear to reject the image of having a non-ideal body and appreciate the image of having an ideal body. In contrast to women, men seem to carry the advantage of being able to rate in a self-serving way if a feature is desirable: If they believe that a body is very desirable, they evaluate it more positively when it belongs to them than when it belongs to someone else. This is in line with studies showing that men evaluate their intelligence as above average, whereas women believe themselves to be of average intelligence (Cooper et al., 2018). Such a gender difference in self-enhancement might contribute to gender differences in ratings both of intelligence and of body dissatisfaction. However, it is conceivable that women do not self-enhance regarding features that are normatively accepted as a weakness in women, such as that women are normatively dissatisfied with their own body (Matthiasdottir et al., 2012), but that they are able to self-enhance regarding features that are stereotypically highly pronounced and important for women, such as being sensitive and caring (Prentice and Carranza, 2002). Future studies should examine which cognitive processes, besides body and intelligence ratings, are influenced by identity in women and men.

Correlation analysis revealed that the higher the degree of body dissatisfaction in women, the less self-serving were the double standards in valence for the overweight body and in body attractiveness for the average-weight body. These results suggest that the higher the degree of body dissatisfaction, the higher the extent of self-deprecating double standards in women, which is line with findings in women with an eating disorder diagnosis, who show a high degree of body dissatisfaction, and were also found to show self-deprecating double standards (Voges et al., 2018). It might be possible that average-weight women with a higher degree of body dissatisfaction reject body fat for themselves to a higher extent than average-weight women with a lower degree of body dissatisfaction (Cafri et al., 2005) insofar as they devalue average-weight and overweight bodies more if the bodies are self-related. In contrast, as body fat is minimal in thin, athletic and hypermuscular bodies, the rejection of body fat might have less influence on the ratings of these bodies. However, further correlations or associations of body dissatisfaction and double standards in men were not found. This might be explained by the fact that in our sample, women as well as men had very low levels of and low variance in body dissatisfaction. To analyze how body dissatisfaction influences the application of double standards in men and women, future studies could examine further samples with higher levels of and higher variance in body dissatisfaction.

Some limitations of the present study should be mentioned. Women and men differed in age, BMI, eating pathology and body dissatisfaction. Therefore, we repeated the analyses with these covariates and added the results as Supplementary Material. Analyses revealed that most of the results remained stable, but some effects changed. To interpret these results, it is important to distinguish expected from unexpected group differences. The group difference in age between men and women was not expected, and might be seen as a random effect of sampling. The effects from the covariance analysis with age did not differ substantially from the analyses without age as a covariate. In contrast, the values and group differences in BMI, eating pathology and body dissatisfaction as occurring in the present study were to be expected, as these variables are related to the grouping variable gender, i.e., men usually have a higher BMI and lower eating pathology and body dissatisfaction than women (Ode et al., 2007; Luce et al., 2008; Quick and Byrd-Bredbenner, 2013; Nevill and Metsios, 2015; Smith et al., 2017). Therefore, a covariance analysis does not only remove variance of the covariate, but also variance of the grouping variable gender. Thus, a covariance analysis does not result in a “pure” effect of gender, but systematically distorts the gender variable (Miller and Chapman, 2001). Following this rational and despite slight changes in significant effects when introducing these covariates (see Supplementary Material), we decided to focus on the results without these covariance analyses in the article, so that conclusions with respect to actual gender differences in body evaluations in the population of younger women and men can be drawn. Furthermore, in order to rule out idiosyncratic differences between the bodies and to create mostly coherent stimuli of faces and bodies, we used body stimuli that were generated by computer software. Therefore, the participants might have perceived the stimuli as artificial. However, as both groups rated the coherence of the stimuli as similarly moderate, a possible decrease in ecological validity would, at least, be equal between the two groups. Furthermore, as we only recruited young adult women and men, the findings might not be generalizable to elderly or younger persons. This issue may be particularly important given that body image changes over the life span (Tiggemann, 2004).

This is the first study to examine double standards in body evaluation in men and to compare these double standards with those applied by women. Men rate moderately or low-attractive bodies more negatively and the ideal body more positively when bodies are presented with their own face compared to another person’s face. In contrast, women are less influenced by identity and mostly rate in a fair-minded manner, with the exception of the overweight body, which led to several self-deprecating double standards for both genders. Thus, we conclude that it may be beneficial for body image research to consider an “identity bias” in body image theory (Williamson et al., 2004) in addition to the already existing biases (e.g., attentional bias, interpretation bias, memory bias) (Voges et al., 2017). Cues that indicate self-relevance (such as the faces in the present study) might activate certain self-related body schemata that influence body rating. This may lead to the emergence of double standards that confirm the self-related body schemata and may be a further factor that maintains the body image of women and men. Female and male stereotypes might impact these processes, leading to gender differences in the application of double standards in body evaluation. Compared to women, for men, it might be advantageous that they are able to self-enhance desirable features, reinforcing their self-worth and body satisfaction.

## Data Availability

The datasets for this manuscript are not publicly available because the local ethics committee of Osnabrück University stipulated that data must not be passed on to third parties. Therefore, data sharing is not applicable to this article.

## Author Contributions

MV planned and conducted the study, analyzed the data, and wrote the first draft of the manuscript. C-MG, MW, AH, and SV contributed to the conception and design of the study. MV, BS, and MW were involved in the development of the stimuli manipulation procedure. All authors contributed to the compilation of the manuscript and read and approved the submitted version.

## Conflict of Interest Statement

The authors declare that the research was conducted in the absence of any commercial or financial relationships that could be construed as a potential conflict of interest.

## Abbreviations

DS score: double standard score
