# Supplementary material for: Gender Differences in Body Evaluation: Do Men Show More Self-Serving Double Standards Than Women?
Source: Front Psychol. 2019 Mar 12;10:544. doi: 10.3389/fpsyg.2019.00544 (PMC6428027; doi:10.3389/fpsyg.2019.00544)
Supplement: Supplementary file 1 [file Table_1.docx]

**Results with age as a covariate:**

**Table 1**

*Means, standard errors, confidence intervals of the means, and post-hoc
t-test results for the double standard scores for each rating variable*

*dependent on the factors Group and Build*

|  | Women | | | Men | | | | Over both  groups | | |
| --- | --- | --- | --- | --- | --- | --- | --- | --- | --- | --- |
| Variables | *M* | *SE* |  | | *M* | *SE* |  | | *M* | *SE* |
| *DS valence* |  |  |  | |  |  |  | |  |  |
| Thin | .091^be^ | .113 |  | | -.300^*af^ | .120 |  | | -.104^e^ | .080 |
| Average-weight | .135^bef^ | .090 |  | | -.346^*af^ | .095 |  | | -.106^e^ | .064 |
| Overweight | -.769^*cdfg^ | .097 |  | | -.557^*fg^ | .102 |  | | -.663^*cdfg^ | .069 |
| Athletic | -.171^bde^ | .088 |  | | .280^*acdeg^ | .093 |  | | .054^e^ | .062 |
| Hypermuscular | -.164^*e^ | .082 |  | | -.070^ef^ | .087 |  | | -.117^*e^ | .058 |
| Over all builds | -.176^*^ | .054 |  | | -.199^*^ | .058 |  | | -.187^*^ | .039 |
| *DS arousal* |  |  |  | |  |  |  | |  |  |
| Thin | .412^*^ | .112 |  | | .720^*^ | .119 |  | | .566^*^ | .080 |
| Average-weight | .423^*^ | .096 |  | | .426^*^ | .102 |  | | .424^*^ | .068 |
| Overweight | 1.039^*^ | .132 |  | | .821^*^ | .140 |  | | .930^*^ | .094 |
| Athletic | .478^*^ | .101 |  | | .530^*^ | .107 |  | | .504^*^ | .072 |
| Hypermuscular | .470^*^ | .104 |  | | .634^*^ | .110 |  | | .552^*e^ | .074 |
| Over all builds | .564^*^ | .079 |  | | .626^*^ | .084 |  | | .595^*^ | .056 |
| *DS body attractiveness* |  |  |  | |  |  |  | |  |  |
| Thin | .031^e^ | .117 |  | | -.127 | .124 |  | | -.048^e^ | .083 |
| Average-weight | -.010^be^ | .089 |  | | -.346^*af^ | .095 |  | | -.178^*^ | .064 |
| Overweight | -.529^*cdfg^ | .079 |  | | -.393^*f^ | .084 |  | | -.461^*^ | .057 |
| Athletic | -.149^be^ | .094 |  | | .203^*adeg^ | .100 |  | | .027^e^ | .067 |
| Hypermuscular | -.149^e^ | .091 |  | | -.257^*f^ | .097 |  | | -.203^*^ | .065 |
| Over all builds | -.161^*^ | .057 |  | | -.184^*^ | .060 |  | | -.173^*^ | .040 |
| *DS body fat* |  |  |  | |  |  |  | |  |  |
| Thin | -.124^*e^ | .060 |  | | -.237^*de^ | .064 |  | | -.181^*^ | .043 |
| Average-weight | .083^be^ | .074 |  | | .441^*acfg^ | .079 |  | | .262^*^ | .053 |
| Overweight | .381^*cdfg^ | .071 |  | | .363^*cfg^ | .075 |  | | .372^*^ | .051 |
| Athletic | -.145^e^ | .082 |  | | -.010^de^ | .087 |  | | -.077 | .058 |
| Hypermuscular | -.062^e^ | .069 |  | | -.030^de^ | .074 |  | | -.046 | .049 |
| Over all builds | .026 | .033 |  | | .105^*^ | .035 |  | | .066^*^ | .023 |
| *DS muscle mass* |  |  |  | |  |  |  | |  |  |
| Thin | -.016 | .078 |  | | -.200^*^ | .083 |  | | -.108 | .055 |
| Average-weight | -.086 | .072 |  | | -.174^*^ | .077 |  | | -.130^*^ | .051 |
| Overweight | -.262^*^ | .062 |  | | -.170^*^ | .066 |  | | -.216^*^ | .044 |
| Athletic | .155^*^ | .068 |  | | .278^*^ | .072 |  | | .217^*^ | .048 |
| Hypermuscular | .068 | .059 |  | | .197^*^ | .062 |  | | .133^*^ | .042 |
| Over all builds | -.028 | .031 |  | | -.014 | .033 |  | | -.021 | .022 |

*Note.* DS = double standard. *M* = Mean. *SE* = standard errors that were used
for calculation of the 95% confidence interval for each DS score.

The MANOVA did not yield a significant main effect of Build.

^*^ zero is out of 95% confidence interval

^a^ differs significantly from women

^b^ differs significantly from men

^c^ differs significantly from the thin build

^d^ differs significantly from the average-weight build

^e^ differs significantly from the overweight build

^f^ differs significantly from the athletic build

^g^ differs significantly from the hypermuscular build

**Results with Body-Mass-Index (BMI) as a covariate:**

**Table 1**

*Means, standard errors, confidence intervals of the means, and post-hoc
t-test results for the double standard scores for each rating variable*

*dependent on the factors Group and Build*

|  | Women | | | Men | | | | Over both  groups | | |
| --- | --- | --- | --- | --- | --- | --- | --- | --- | --- | --- |
| Variables | *M* | *SE* |  | | *M* | *SE* |  | | *M* | *SE* |
| *DS valence* |  |  |  | |  |  |  | |  |  |
| Thin | -.022^e^ | .131 |  | | -.173^f^ | .141 |  | | -.098 | .080 |
| Average-weight | .263^*befg^ | .103 |  | | -.490^*af^ | .111 |  | | -.113 | .063 |
| Overweight | -.752^*cdfg^ | .112 |  | | -.576^*fg^ | .121 |  | | -.664^*^ | .069 |
| Athletic | -.152^bde^ | .102 |  | | .258^*adeg^ | .110 |  | | .053 | .063 |
| Hypermuscular | -.118^de^ | .095 |  | | -.122^ef^ | .102 |  | | -.120^*^ | .058 |
| Over all builds | -.156^*^ | .063 |  | | -.221^*^ | .068 |  | | -.188^*^ | .039 |
| *DS arousal* |  |  |  | |  |  |  | |  |  |
| Thin | .411^*^ | .131 |  | | .721^*^ | .141 |  | | .566^*^ | .080 |
| Average-weight | .431^*^ | .111 |  | | .417^*^ | .120 |  | | .424^*^ | .068 |
| Overweight | .998^*^ | .154 |  | | .867^*^ | .165 |  | | .932^*^ | .094 |
| Athletic | .538^*^ | .117 |  | | .463^*^ | .126 |  | | .500^*^ | .072 |
| Hypermuscular | .510^*^ | .121 |  | | .589^*^ | .130 |  | | .549^*^ | .074 |
| Over all builds | .577^*^ | .092 |  | | .611^*^ | .098 |  | | .594^*^ | .056 |
| *DS body attractiveness* |  |  |  | |  |  |  | |  |  |
| Thin | -.090 | .137 |  | | .008^d^ | .148 |  | | -.041 | .084 |
| Average-weight | .120^be^ | .103 |  | | -.491^*cf^ | .110 |  | | -.186^*^ | .063 |
| Overweight | -.493^*dfg^ | .092 |  | | -.433^*f^ | .099 |  | | -.463^*^ | .057 |
| Athletic | -.039^e^ | .109 |  | | .081^deg^ | .117 |  | | .021 | .067 |
| Hypermuscular | -.105^e^ | .106 |  | | -.306^*f^ | .114 |  | | -.206^*^ | .065 |
| Over all builds | -.122 | .066 |  | | -.228^*^ | .070 |  | | -.175^*^ | .040 |
| *DS body fat* |  |  |  | |  |  |  | |  |  |
| Thin | -.146^*e^ | .070 |  | | -.213^*de^ | .075 |  | | -.180^*^ | .043 |
| Average-weight | -.002^be^ | .085 |  | | .435^*acfg^ | .092 |  | | .267^*^ | .052 |
| Overweight | .352^*cdfg^ | .083 |  | | .395^*cg^ | .089 |  | | .374^*^ | .051 |
| Athletic | -.175^e^ | .097 |  | | .024^d^ | .104 |  | | -.076 | .059 |
| Hypermuscular | -.122^e^ | .080 |  | | .037^de^ | .086 |  | | -.042 | .049 |
| Over all builds | -.018 | .037 |  | | .156^*^ | .040 |  | | .069^*^ | .023 |
| *DS muscle mass* |  |  |  | |  |  |  | |  |  |
| Thin | -.106 | .090 |  | | -.099 | .097 |  | | -.102 | .055 |
| Average-weight | -.082 | .084 |  | | -.179 | .091 |  | | -.131^*^ | .052 |
| Overweight | -.268^*^ | .072 |  | | -.163^*^ | .078 |  | | -.215^*^ | .044 |
| Athletic | .191^*^ | .080 |  | | .238^*^ | .086 |  | | .214^*^ | .049 |
| Hypermuscular | .096 | .068 |  | | .166^*^ | .073 |  | | .131^*^ | .042 |
| Over all builds | -.034 | .037 |  | | -.007 | .040 |  | | -.021 | .023 |

*Note.* DS = double standard. *M* = Mean. *SE* = standard errors that were used
for calculation of the 95% confidence interval for each DS score.

The MANOVA did not yield a significant main effect of Build.

^*^ zero is out of 95% confidence interval

^a^ differs significantly from women

^b^ differs significantly from men

^c^ differs significantly from the thin build

^d^ differs significantly from the average-weight build

^e^ differs significantly from the overweight build

^f^ differs significantly from the athletic build

^g^ differs significantly from the hypermuscular build

**Results with Eating pathology (EDE-Q score) as a covariate:**

**Table 1**

*Means, standard errors, confidence intervals of the means, and post-hoc
t-test results for the double standard scores for each rating variable*

*dependent on the factors Group and Build*

|  | Women | | | Men | | | | Over both  groups | | |
| --- | --- | --- | --- | --- | --- | --- | --- | --- | --- | --- |
| Variables | *M* | *SE* |  | | *M* | *SE* |  | | *M* | *SE* |
| *DS valence* |  |  |  | |  |  |  | |  |  |
| Thin | .074^be^ | .112 |  | | -.280^*af^ | .118 |  | | -.103^e^ | .080 |
| Average-weight | .174^*befg^ | .087 |  | | -.391^*afg^ | .092 |  | | -.108^e^ | .063 |
| Overweight | -.745^*cdfg^ | .095 |  | | -.584^*fg^ | .101 |  | | -.665^*cdfg^ | .068 |
| Athletic | -.182^*bde^ | .086 |  | | .291^*acdeg^ | .092 |  | | .055^e^ | .062 |
| Hypermuscular | -.184^*de^ | .080 |  | | -.049^def^ | .085 |  | | -.116^*e^ | .058 |
| Over all builds | -.172^*^ | .053 |  | | -.202^*^ | .057 |  | | -.187^*^ | .038 |
| *DS arousal* |  |  |  | |  |  |  | |  |  |
| Thin | .437^*^ | .111 |  | | .692^*^ | .118 |  | | .564^*e^ | .080 |
| Average-weight | .396^*^ | .094 |  | | .456^*^ | .100 |  | | .426^*e^ | .068 |
| Overweight | .987^*^ | .129 |  | | .879^*^ | .137 |  | | .933^*cdfg^ | .093 |
| Athletic | .487^*^ | .100 |  | | .520^*^ | .106 |  | | .503^*e^ | .072 |
| Hypermuscular | .459^*^ | .103 |  | | .646^*^ | .109 |  | | .552^*e^ | .074 |
| Over all builds | .553^*^ | .078 |  | | .639^*^ | .082 |  | | .596^*^ | .056 |
| *DS body attractiveness* |  |  |  | |  |  |  | |  |  |
| Thin | .010^e^ | .116 |  | | -.103 | .123 |  | | -.047^e^ | .084 |
| Average-weight | .035^be^ | .087 |  | | -.396^*af^ | .092 |  | | -.181^*e^ | .062 |
| Overweight | -.519^*cdfg^ | .079 |  | | -.405^*f^ | .083 |  | | -.462^*cdfg^ | .057 |
| Athletic | -.175^be^ | .094 |  | | .232^*adeg^ | .099 |  | | .029^eg^ | .068 |
| Hypermuscular | -.155^e^ | .090 |  | | -.250^*f^ | .095 |  | | -.203^*ef^ | .065 |
| Over all builds | -.161^*^ | .056 |  | | -.184^*^ | .059 |  | | -.173^*^ | .040 |
| *DS body fat* |  |  |  | |  |  |  | |  |  |
| Thin | -.122^*e^ | .060 |  | | -.240^*de^ | .063 |  | | -.181^*de^ | .043 |
| Average-weight | .067^be^ | .072 |  | | .458^*acfg^ | .077 |  | | .263^*cfg^ | .052 |
| Overweight | .375^*cdfg^ | .070 |  | | .370^*cfg^ | .074 |  | | .372^*cfg^ | .051 |
| Athletic | -.122^e^ | .082 |  | | -.036^de^ | .087 |  | | -.079^de^ | .059 |
| Hypermuscular | -.071^e^ | .069 |  | | -.019^de^ | .073 |  | | -.045^de^ | .049 |
| Over all builds | .025 | .032 |  | | .107^*^ | .034 |  | | .066^*^ | .023 |
| *DS muscle mass* |  |  |  | |  |  |  | |  |  |
| Thin | -.040 | .077 |  | | -.172^*^ | .082 |  | | -.106^fg^ | .055 |
| Average-weight | -.084 | .071 |  | | -.177 | .075 |  | | -.131^*fg^ | .051 |
| Overweight | -.249^*^ | .061 |  | | -.184^*^ | .065 |  | | -.216^*fg^ | .044 |
| Athletic | .110 | .069 |  | | .329^*^ | .073 |  | | .219^*cde^ | .049 |
| Hypermuscular | .075 | .057 |  | | .189^*^ | .061 |  | | .132^*cde^ | .041 |
| Over all builds | -.038 | .031 |  | | -.003 | .033 |  | | -.020 | .023 |

*Note.* DS = double standard. *M* = Mean. *SE* = standard errors that were used
for calculation of the 95% confidence interval for each DS score.

^*^ zero is out of 95% confidence interval

^a^ differs significantly from women

^b^ differs significantly from men

^c^ differs significantly from the thin build

^d^ differs significantly from the average-weight build

^e^ differs significantly from the overweight build

^f^ differs significantly from the athletic build

^g^ differs significantly from the hypermuscular build

**Results with Body dissatisfaction (subscale EDI-2) as a covariate:**

**Table 1**

*Means, standard errors, confidence intervals of the means, and post-hoc
t-test results for the double standard scores for each rating variable*

*dependent on the factors Group and Build*

|  | Women | | | Men | | | | Over both  groups | | |
| --- | --- | --- | --- | --- | --- | --- | --- | --- | --- | --- |
| Variables | *M* | *SE* |  | | *M* | *SE* |  | | *M* | *SE* |
| *DS valence* |  |  |  | |  |  |  | |  |  |
| Thin | .078^be^ | .114 |  | | -.285^*af^ | .114 |  | | -.104^e^ | .080 |
| Average-weight | .180^*befg^ | .089 |  | | -.397^*afg^ | .095 |  | | -.109^e^ | .063 |
| Overweight | -.717^*cdfg^ | .096 |  | | -.615^*fg^ | .102 |  | | -.666^*cdfg^ | .068 |
| Athletic | -.146^bde^ | .087 |  | | .251^*acdeg^ | .092 |  | | .053^e^ | .061 |
| Hypermuscular | -.167^*de^ | .082 |  | | -.067^def^ | .087 |  | | -.117^*e^ | .058 |
| Over all builds | -.154^*^ | .054 |  | | -.223^*^ | .057 |  | | -.189^*^ | .038 |
| *DS arousal* |  |  |  | |  |  |  | |  |  |
| Thin | .470^*^ | .112 |  | | .655^*^ | .119 |  | | .563^*e^ | .080 |
| Average-weight | .385^*^ | .096 |  | | .469^*^ | .102 |  | | .427^*e^ | .068 |
| Overweight | .981^*^ | .132 |  | | .886^*^ | .140 |  | | .933^*cdfg^ | .094 |
| Athletic | .482^*^ | .102 |  | | .525^*^ | .108 |  | | .504^*e^ | .072 |
| Hypermuscular | .437^*^ | .104 |  | | .670^*^ | .110 |  | | .554^*e^ | .074 |
| Over all builds | .551^*^ | .079 |  | | .641^*^ | .084 |  | | .596^*^ | .056 |
| *DS body attractiveness* |  |  |  | |  |  |  | |  |  |
| Thin | .003^e^ | .119 |  | | -.096 | .126 |  | | -.046^e^ | .084 |
| Average-weight | .050^be^ | .088 |  | | -.413^*af^ | .094 |  | | -.181^*e^ | .063 |
| Overweight | -.528^*cdfg^ | .080 |  | | -.395^*f^ | .085 |  | | -.461^*cdfg^ | .057 |
| Athletic | -.155^be^ | .095 |  | | .209^*adeg^ | .101 |  | | .027^eg^ | .067 |
| Hypermuscular | -.150^e^ | .092 |  | | -.256^*f^ | .097 |  | | -.203^*ef^ | .065 |
| Over all builds | -.156^*^ | .057 |  | | -.190^*^ | .060 |  | | -.173^*^ | .040 |
| *DS body fat* |  |  |  | |  |  |  | |  |  |
| Thin | -.131^*e^ | .061 |  | | -.230^*de^ | .064 |  | | -.180^*de^ | .043 |
| Average-weight | .068^be^ | .074 |  | | .457^*acfg^ | .079 |  | | .263^*cfg^ | .052 |
| Overweight | .366^*cdfg^ | .071 |  | | .380^*cfg^ | .076 |  | | .373^*cfg^ | .051 |
| Athletic | -.122^e^ | .084 |  | | -.035^de^ | .089 |  | | -.079^de^ | .059 |
| Hypermuscular | -.044^e^ | .069 |  | | -.050^de^ | .074 |  | | -.047^de^ | .049 |
| Over all builds | .027 | .033 |  | | .104^*^ | .035 |  | | .066^*^ | .023 |
| *DS muscle mass* |  |  |  | |  |  |  | |  |  |
| Thin | -.045 | .078 |  | | -.167^*^ | .083 |  | | -.106^fg^ | .055 |
| Average-weight | -.073 | .072 |  | | -.189^*^ | .077 |  | | -.131^*fg^ | .051 |
| Overweight | -.258^*^ | .062 |  | | -.174^*^ | .066 |  | | -.216^*fg^ | .044 |
| Athletic | .109 | .070 |  | | .329^*^ | .074 |  | | .219^*cde^ | .049 |
| Hypermuscular | .060 | .059 |  | | .206^*^ | .062 |  | | .133^*cde^ | .042 |
| Over all builds | -.041 | .032 |  | | .001 | .034 |  | | -.020 | .023 |

*Note.* DS = double standard. *M* = Mean. *SE* = standard errors that were used
for calculation of the 95% confidence interval for each DS score.

^*^ zero is out of 95% confidence interval

^a^ differs significantly from women

^b^ differs significantly from men

^c^ differs significantly from the thin build

^d^ differs significantly from the average-weight build

^e^ differs significantly from the overweight build

^f^ differs significantly from the athletic build

^g^ differs significantly from the hypermuscular build
